# High-flow nasal cannula failure in critically ill cancer patients with acute respiratory failure: Moving from avoiding intubation to avoiding delayed intubation

**DOI:** 10.1371/journal.pone.0270138

**Published:** 2022-06-29

**Authors:** Colombe Saillard, Jérôme Lambert, Morgane Tramier, Laurent Chow-Chine, Magali Bisbal, Luca Servan, Frederic Gonzalez, Jean-Manuel de Guibert, Marion Faucher, Antoine Sannini, Djamel Mokart

**Affiliations:** 1 Hematology Department, Institut Paoli Calmettes, Marseille, France; 2 Biostatistics Unit, INSERM U1153, Hopital Saint Louis, Paris Diderot University, Paris, France; 3 Department of Anesthesiology and Critical Care, Polyvalent Intensive Care Unit, Institut Paoli Calmettes, Marseille, France; University Hospital of Modena (Italy), Respiratory Diseases Unit, ITALY

## Abstract

**Background:**

High-flow nasal cannula (HFNC) is increasingly used in critically ill cancer patients with acute respiratory failure (ARF) to avoid mechanical ventilation (MV). The objective was to assess prognostic factors associated with mortality in ICU cancer patients requiring MV after HFNC failure, and to identify predictive factors of intubation.

**Methods:**

We conducted a retrospective study from 2012–2016 in a cancer referral center. All consecutive onco-hematology adult patients admitted to the ICU treated with HFNC were included. HFNC failure was defined by intubation requirement.

**Results:**

202 patients were included, 104 successfully treated with HFNC and 98 requiring intubation. ICU and hospital mortality rates were 26.2% (n = 53) and 42.1% (n = 85) respectively, and 53.1% (n = 52) and 68.4% (n = 67) in patients requiring MV. Multivariate analysis identified 4 prognostic factors of hospital mortality after HFNC failure: complete/partial remission (OR = 0.2, 95%CI = 0.04–0.98, p<0.001) compared to patients with refractory/relapse disease (OR = 3.73, 95%CI = 1.08–12.86), intubation after day 3 (OR = 7.78, 95%CI = 1.44–41.96), number of pulmonary quadrants involved on chest X-ray (OR = 1.93, 95%CI = 1.14–3.26, p = 0.01) and SAPSII at ICU admission (OR = 1.06, 95%CI = 1–1.12, p = 0.019). Predictive factors of intubation were the absence of sepsis (sHR = 0.32, 95%CI = 0.12–0.74, p = 0.0087), Sp02<95% 15 minutes after HFNC initiation (sHR = 2.05, 95%CI = 1.32–3.18, p = 0.0014), number of quadrants on X-ray (sHR = 1.73, 95%CI = 1.46–2.06, p<0.001), Fi02>60% at HFNC initiation (sHR = 3.12, 95%CI = 2.06–4.74, p<0.001) and SAPSII at ICU admission (sHR = 1.03, 95%CI = 1.02–1.05, p<0.01).

**Conclusion:**

Duration of HFNC may be predictive of an excess mortality in ARF cancer patients. Early warning scores to predict HFNC failure are needed to identify patients who would benefit from early intubation.

## Introduction

Acute respiratory failure (ARF) is a frequent and life-threatening complication in immunocompromised patients, raising major diagnostic and therapeutic challenges. It occurs in up to half of patients with hematological malignancies and 15% of patients with solid tumors and represents the first cause for intensive care (ICU) admission in cancer patients. Mortality can reach 50%, depending on underlying condition, etiology, severity and course of ARF, delayed ICU admission, need for mechanical ventilation (MV), and associated organ dysfunctions at ICU admission [1–5]

Initial management of ARF consists of optimizing oxygenation, identifying ARF etiology guided by a standardized diagnostic approach [3] and supporting associated organ dysfunction at the same time [6]. The optimal ventilation strategy in cancer patients with ARF remains controversial [7]. Considering the mortality rates in patients requiring MV, non-invasive ventilation strategies have been recently widely evaluated, priority has been given to avoid intubation [8]. Non-invasive ventilation (NIV) was first investigated with significant reduction in intubation and mortality rates [9], but challenged by larger and multicenter data [2, 10].

High-flow nasal cannula (HFNC) oxygen therapy delivers warm and humidified oxygen through a nasal cannula, allowing for airflows as high as 50 liters/minute to achieve inspired oxygen fractions (FiO2) as high as 100% [11, 12]. It has been increasingly used recently [13]. In unselected patients with ARF, HFNC was associated with increased ventilator-free days and decreased day-90 mortality [14]. In two recent meta-analyses, HFNC may decrease the need for tracheal intubation without impacting mortality [15, 16]. Uncertainty remains about HFNC effects in immunocompromised patients, studies providing conflicting results. Recent data did not find any significant survival or clinical benefit compared with standard oxygen [10, 14, 17], whereas other publications demonstrated that HFNC may decrease intubation requirement and/or mortality [2, 18, 19]. The absence of diagnosis and mechanical ventilation requirement are the main prognostic factors [2].

For patients who fail to improve with HFNC, intubation should be strongly considered. HFNC failure in immunocompromised patients has been rarely investigated. The objective of our study was to assess prognostic factors of mortality in ICU cancer patients with ARF requiring MV after HFNC failure, and to identify predictive factors of intubation.

## Material and methods

### Patients’ selection

We conducted a retrospective study from 2012–2016 in our institution (Paoli-Calmettes Institute, Marseille, France), a cancer referral center. All adult patients with solid tumor or hematological malignancy admitted to the ICU for ARF treated with HFNC requiring oxygen ≥30 L/minute were included. Exclusion criteria’s were acute cardiogenic pulmonary edema, hypercapnic ARF, MV weaning, HFNC during scheduled surgery, treatment-limitation with do-not-intubate decision and patients intubated after HFNC weaning at ICU admission. The study was approved by our local Institutional Review Board.

### HFNC device

All patients received high-flow oxygen therapy via HFNC device (Optiflow, Fisher & Paykel Healthcare, Auckland, New Zealand). Oxygen was applied continuously by large-bore binasal prongs at a gas flow of 50 L/min with a FiO2 level of 100% initially through a heated humidifier (MR850, Fisher&Paykel Healthcare). Response to treatment was continuously monitored. The aim of oxygenation was to produce peripheral capillary oxygen saturation (SpO2) levels of 92% or more. Patients were treated with complementary non-invasive ventilation according to the intensivist in charge according to local guidelines [19].

### Definitions and data collection

Demographical, clinical, biological and outcome data were retrospectively collected from the patient’s charts using our ICU management software (MetavisionTM, Dusseldorf, Germany). We recorded the following baseline data at ICU admission and during ICU stay: age, gender, underlying malignancy, allogenic or autologous hematopoietic stem cell transplantation (HSCT), disease status, neutropenia, Charlson comorbidities index [20]. Severity of illness was assessed using Simplified Acute Physiology Score (SAPS 2) [21] and Sepsis-related Organ Failure Assessment (SOFA) score [22].

ARF was defined as a need for oxygen greater than 6L/min to maintain peripheral capillary oxygen saturation >95% or symptoms of respiratory distress (tachypnea >30/min, intercostal recession, labored breathing, and/or dyspnea at rest). Neutropenia was defined by absolute neutrophil count < 0.5 G/L. We quantified the number of involved pathologic quadrants on chest X-ray (0–4) at ICU admission [23]. Fever was classified as clinically documented, microbiologically documented or fever of unknown origin. HFNC settings and respiratory function were collected during ICU stay. HFNC failure was defined by intubation and MV requirement. Criteria for endotracheal intubation included signs of persisting or worsening respiratory failure (defined as two of the following criteria: respiratory rate above 40 breaths per min, lack of improvement in signs of high respiratory-muscle workload, development of copious tracheal secretions, pH <7.35, SpO2 levels <90% for more than 5 min without technical dysfunction, or intolerance to oxygenation techniques), hemodynamic instability (systolic blood pressure <90 mm Hg, mean blood pressure <65 mm Hg, or vasopressors) or neurological deterioration (Glasgow<12) [14]. Outcome endpoints including intubation rate, length of HFNC, time from HFNC initiation to MV initiation (at day 0 (≤ 24 hours), day 1, day 2–3 and > day 3), ICU, hospital and one-year mortality.

### Statistical analysis

All data are presented as rates (percentage) for qualitative variables and medians (25th-75th percentiles) for quantitative variables. Characteristics of patients subsequently intubated were compared across the groups of hospital survivors and non-survivors by using Fisher’s exact test and Wilcoxon rank-sum test. We performed logistic regression analyses to identify variables independently associated with hospital mortality, as measured by the estimated odds ratio (OR) with 95% confidence interval (95% CI). Variables yielding *p* lower than 0.15 in the bivariable analyses were entered into a multivariable logistic regression model with hospital mortality as the outcome and a stepwise forward variable selection.

In the second part, we analyzed all patients treated with HFNC in order to identify predictive factors of intubation. Similarly, we compared characteristics of patients according to HFNC success (no intubation) or failure (intubation) by using bivariable Fine and Gray model which accounts for the competing risk of discharge form ICU without intubation. All variables associated with intubation at a p-value lower than 0.15 were then included in a multivariable Fine and Gray regression model with stepwise forward variable selection. Association between covariate and risk of intubation should have been reported as subdistribution Hazard Ratio (sHR). All tests were two-sided, and *p* values lower than 0.05 were considered statistically significant. Statistical tests were conducted using the SPSS 13 software package (IBM, Armonk, NY, USA) and R software (R Core Team, 2020).

## Results

**Fig 1:**
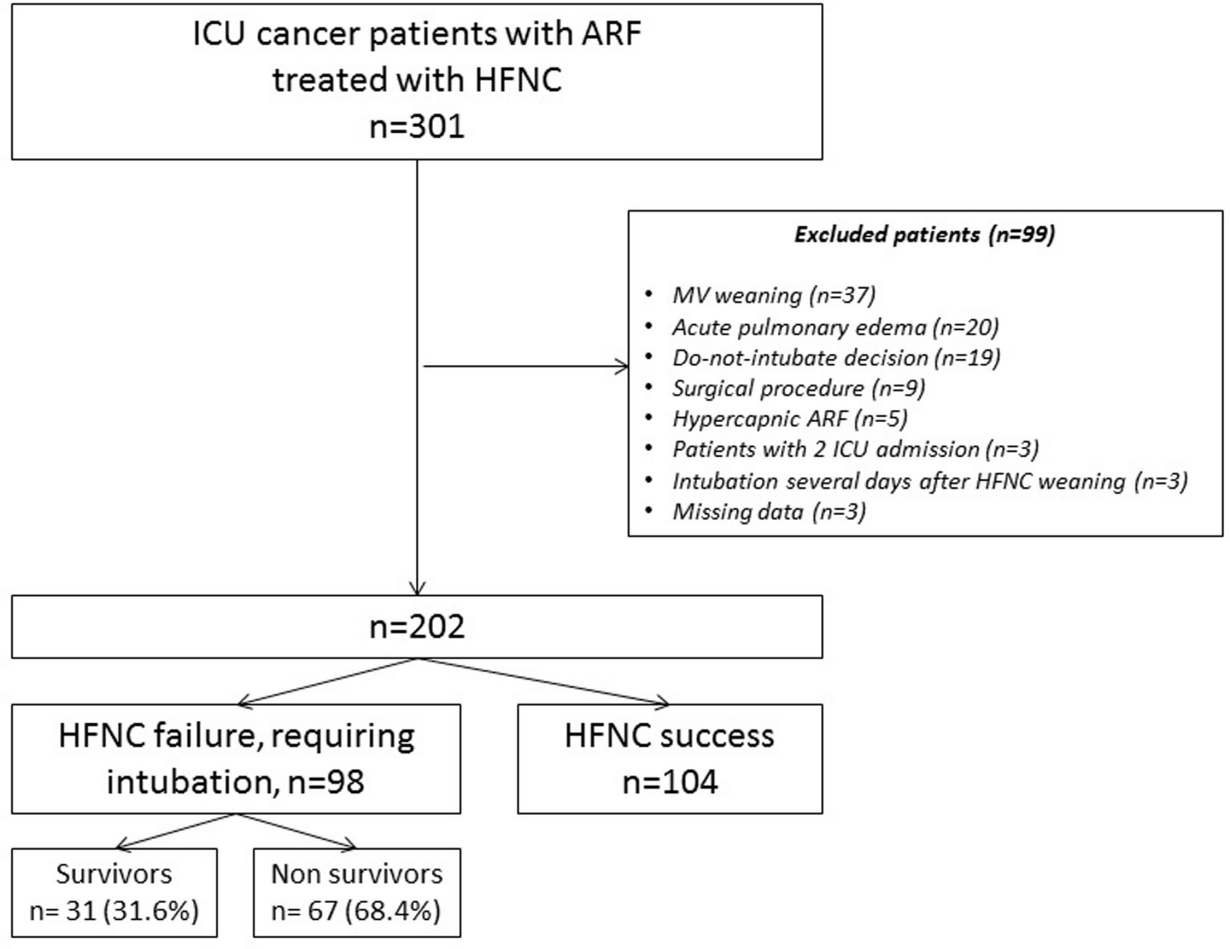
Flow chart of selected patients.

During the study period, 202 cancer patients with ARF treated with HFNC were included (Fig 1). 104 patients were successfully treated with HFNC and 98 patients experienced HFNC failure and required subsequent intubation and MV.

In the 202 included patients, ICU and hospital mortality rates were 26.2% (n = 53) and 42.1% (n = 85) respectively. In patients requiring intubation (n = 98), ICU and hospital mortality rates were 53.1% (n = 52) and 68.4% (n = 67) respectively. In patients treated successfully with HFNC (n = 104), ICU and hospital mortality rates were 1% (n = 1) and 17.3% (n = 18) respectively.

### Characteristics of patients intubated

**Fig 2:**
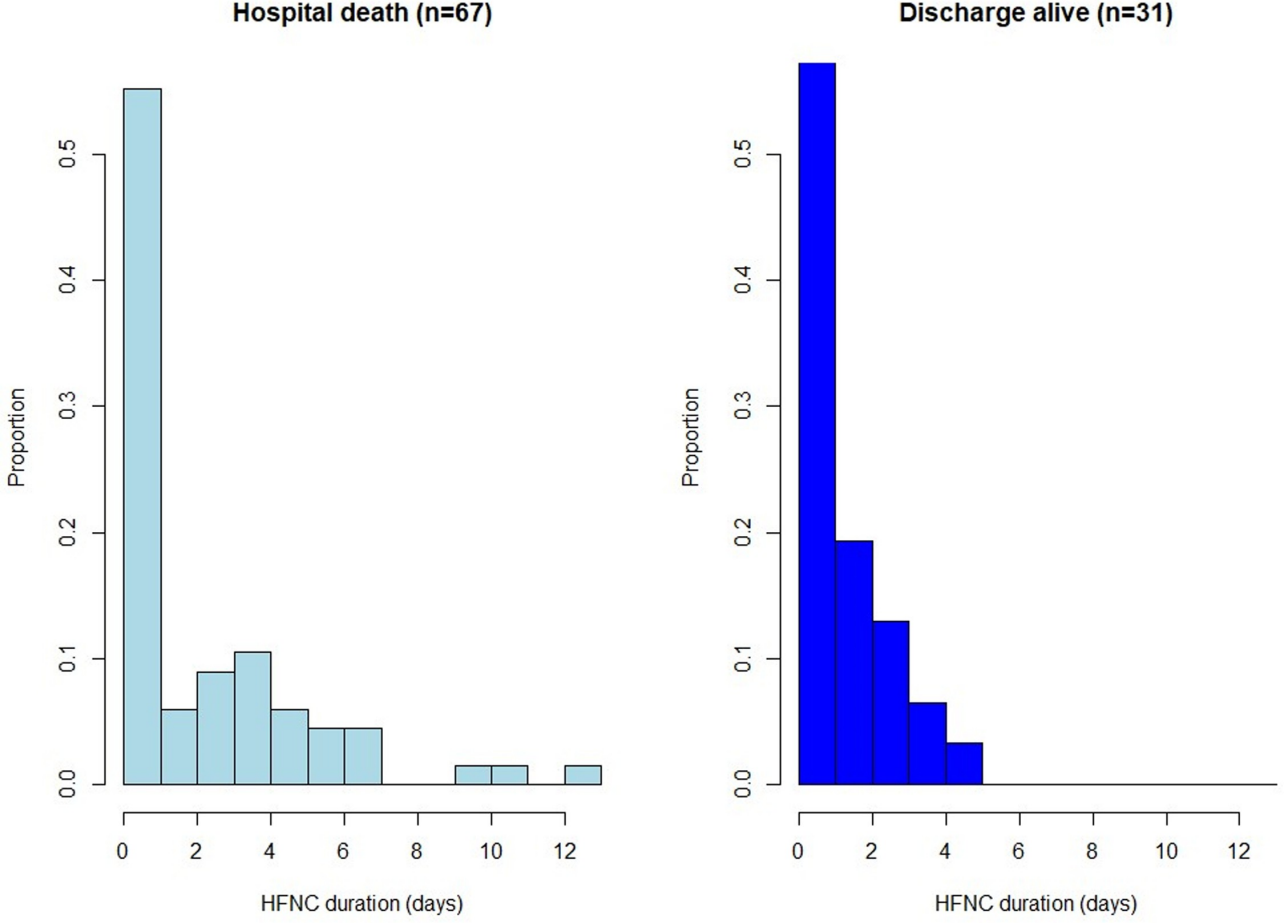
Hospital mortality and survival according to duration of HFNC (days).

**Fig 3:**
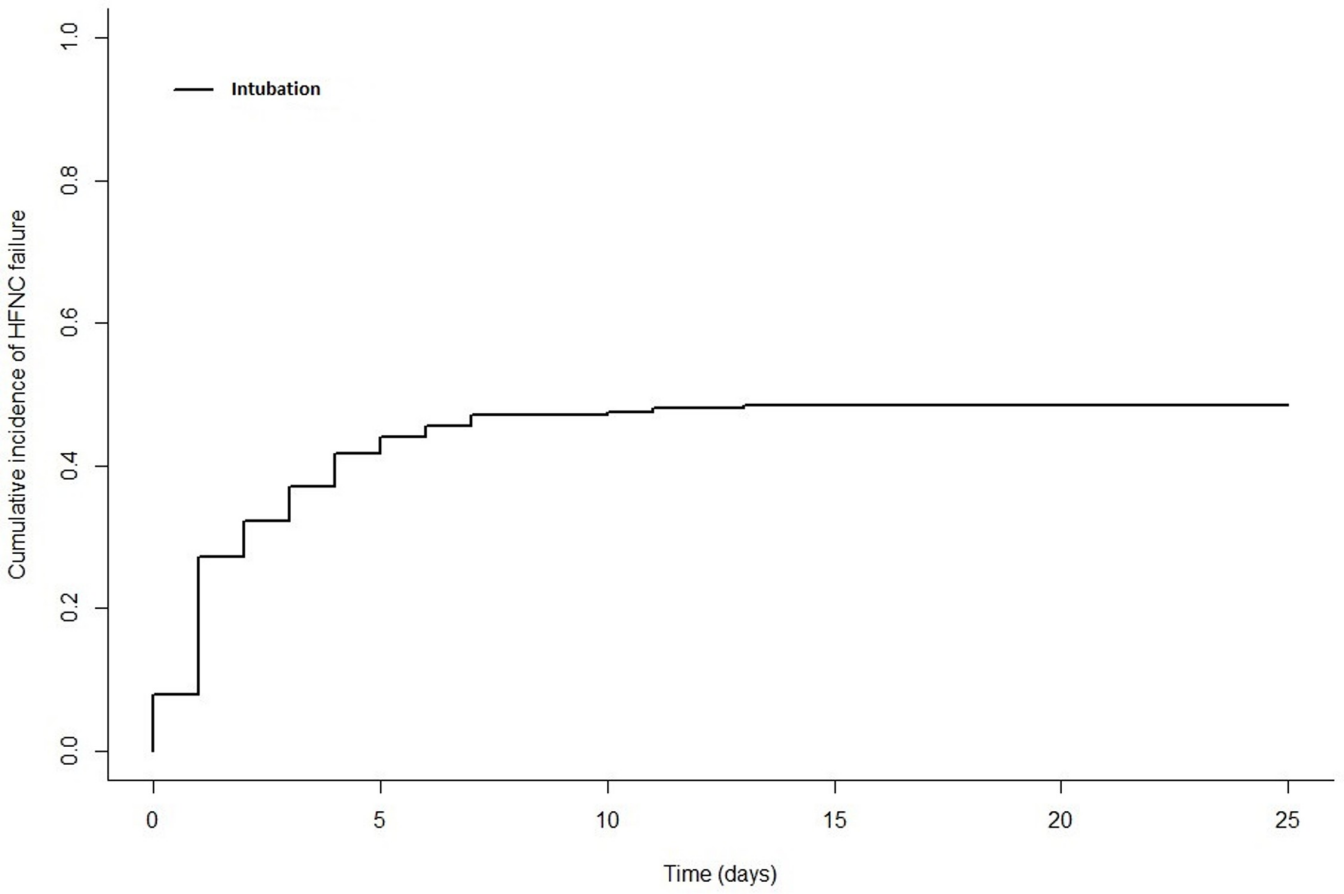
Cumulative incidence of intubation in days.

In the 98 patients who required MV, median age was 62.5 (54–68.7) (Table 1). Underlying malignancies were mainly acute leukemias (33.7%), lymphomas (21.4%), chronic leukemias (5.1%) and solid tumors (26.5%). Cancer was newly diagnosed in 39.8%, in progression or relapse in 44.9% and in remission in 15.3%. Allogenic HSCT patients represented 23.5% of patients. Patients were neutropenic at ICU admission in 25.8%. Median SAPS II and SOFA scores at ICU admission were 43 (37–50) and 5 (2–7) respectively. At HFNC initiation, median respiratory rate were 28 (21–34) and 3 (2–4) quadrants were involved on chest X-ray. Median Fi02 and oxygen flow were 70% (50–100) and 50 L/min (40–50) respectively. Saturation of oxygen and respiratory rate 15 minutes after HFNC initiation were 96% (93–98) and 25 (17–33) respectively. At intubation, median SOFA score was 8 (6–11), with 3 (2–4) pulmonary quadrants involved. In patients requiring MV, HFNC failure was observed in the first 24 hours (day 0) (16.3%), at day 1 (39.8%), at day 2 and 3 (20.4%) or after day 3 (23.5%). Fig 2 illustrates hospital mortality and survival according to HFNC duration (Fig 2). Other organ support consisted of NIV in 42.9%, renal replacement therapy in 29.6% and vasopressors in 93.9% of patients. Patients had a bacterial documented sepsis in 36.7%, including multi-drug bacteria in 17.3%, viral documentation in 28.6% and fungal in 20.4%. Fig 3 represented cumulative incidence of intubation (Fig 3).

**Table 1:** Characteristics of patients experiencing HFNC therapy failure.

	All patients n = 98	Hospital survivors n = 31 (31.6%)	Hospital non survivors n = 67 (68.4%)	*p*
Female gender	63 (64.3%)	23 (74.2%)	40 (59.7%)	0.18
Age (years)	62.5 [54–68.7]	60 [52.5–68]	63 [55.5–69]	0.36
BMI	23.6 [21.4–25.8]	24.9 [21.3–26.3]	23.3 [21.6–25.7]	0.87
Charlson comorbidity index	4 [3–6]	4 [3–5]	4 [3–6]	0.36
Main underlying malignancy				0.35
Acute leukemias	33 (33.7%)	8 (25.8%)	25 (37.3%)	
Lymphomas	21 (21.4%)	8 (25.8%)	13 (19.4%)	
CLL/CML	5 (5.1%)	2 (6.5%)	3 (4.5%)	
Other hematology diseases	13 (13.3%)	2 (6.5%)	11 (16.4%)	
Solid tumors	26 (26.5%)	11 (35.5%)	15 (22.4%)	
Allo-HSCT	23 (23.5%)	5 (16.1%)	18 (26.9%)	0.3
Neutropenia at ICU admission	25 (25.8%)	7 (22.6%)	18 (27.3%)	0.8
Disease status				**0.003**
Newly diagnosed	39 (39.8%)	15 (48.4%)	24 (35.8%)	
Progression/Relapse	44 (44.9%)	7 (22.6%)	37 (55.2%)	
Complete or partial response	15 (15.3%)	9 (29%)	6 (9%)	
At ICU admission				
SAPS II	43 [37–50]	40.5 [32–45.7]	44 [37–53.5]	**0.03**
SOFA score	5 [2–7]	4 [2–7]	5 [3–8]	**0.007**
At HFNC initiation				
SOFA score	5 [3–8]	5 [2–7]	6 [4–8]	**0.003**
Respiratory SOFA: Pa02/Fi02				0.80
>400	1 (1%)	0	1 (1.5%)	
301–400	8 (8.2%)	3 (9.7%)	5 (7.5%)	
≤300	89 (90.1%)	28 (90.3%)	61 (91%)	
Breath rate at the onset (/min)	28 [21–34]	28 [22–34.5]	26 [20.2–33.7]	0.69
Nb of quadrants on chest-X-rays	3 [2–4]	3 [1.5–3.5]	3 [2–4]	0.04
FiO2 (%)	70 [50–100]	70 [50–90]	70 [60–100]	0.057
Oxygen flow (L/min)	50 [40–50]	50 [40–50]	50 [40–50]	0.35
After HFNC initiation				
Breath rate 15 min later	25 [17–33]	22 [18–32]	26 [16–33]	0.72
SpO2 15 min later (%)	96 [93–98]	96 [94.5–98]	95 [93–98]	0.52
At intubation				
SOFA score	8 [6–11]	7 [5–9.5]	8 [6–12]	0.1
Breath rate at the onset	35[27–39]	35[27–38]	34[26–40]	0.97
Nb of quadrants on chest-X-rays	3 [2–4]	3 [2–4]	4 [2–4]	0.18
Duration of HFNC prior intubation				**0.05**
From ICU admission ≤ 24h (D0)	16 (16.3%)	4 (12.9%)	12 (17.9%)	
≤ 48 hours (Day 1)	39 (39.8%)	14 (45.2%)	25 (37.3%)	
From Day 2 to Day 3	20 (20.4%)	10 (32.3%)	10 (14.9%)	
After Day 3	23 (23.5%)	3 (9.7%)	20 (29.9%)	
Support during ICU stay				
NIV	42(42.9%)	12 (38.7%)	30 (44.8%)	0.573
Renal replacement therapy	29(29.6%)	6 (19.4%)	23 (34.3%)	0.131
Vasopressors	92(93.9%)	27(87.1%)	65(97%)	0.057
Documented sepsis (all sepsis)				
Bacterial	36 (36.7%)	12 (38.7%)	24 (35.8%)	0.82
Including MDR bacteria	17 (17.3%)	2 (6.5%)	15 (22.4%)	0.08
Fungal	20 (20.4%)	4 (12.9%)	16 (23.9%)	0.28
Viral	28 (28.6%)	8 (25.8%)	20 (29.9%)	0.8
Fever of unknown origin	15 (15.3%)	5 (16.1%)	10 (14.9%)	1
Absence of sepsis	5 (5.1%)	2 (6.5%)	3 (4.5%)	0.65

AlloHSCT: allogenic hematopoietic stem cell transplantation, BMI: body mass index, CLL: chronic lymphocytic leukemia, CML: chronic myeloid leukemia, Fi02: fraction of inspired oxygen, HFNC: high flow nasal cannula, ICU: intensive care unit, NIV: non-invasive ventilation, MDR: multi-drug resistant, SAPS II: simplified acute physiology score II, SOFA: sequential organ failure assessment, Sp02: oxygen saturation.

### Prognostic factors of hospital mortality of intubated patients

Multivariate analysis identified 4 prognostic factors of hospital mortality after HFNC failure. Complete or partial remission had a favorable prognostic impact (OR = 0.2, 95% CI = 0.04–0.98, p<0.001) compared to patients with refractory or relapse disease (OR = 3.73, 95% CI = 1.08–12.86). Late intubation was predictive of unfavorable outcome, patients intubated after day 3 carrying a significantly higher hospital mortality (OR = 7.78, 95% CI = 1.44–41.96) compared to early intubation (p = 0.017). The number of pulmonary quadrants involved on chest X-ray was prognostic of increased hospital mortality (OR = 1.93, 95% CI = 1.14–3.26, p = 0.01), as well as SAPSII at ICU admission (OR = 1.06, 95% CI = 1–1.12, p = 0.019) (Table 2).

**Table 2:** Multivariate analysis of prognostic factors associated with hospital mortality in patients with HFNC failure.

	OR	95% CI	*p*
Disease status			
Diagnosis	1		0.00075
Refractory or relapse	3.73	[1.08–12.86]	
Remission (complete or partial)	0.2	[0.04–0.98]	
Number of pulmonary quadrants on chest X-ray	1.93	[1.14–3.26]	0.01
Duration of HFNC before intubation			
Day 1	1		0.017
≤ 24 hours (Day 0)	1.7	[0.3–9.75]	
Day 2 and 3	0.65	[0.17–2.42]	
> Day 3	7.78	[1.44–41.96]	
SAPS II at ICU admission	1.06	[1–1.12]	0.019

CI: confidence interval, HFNC: high flow nasal cannula, ICU: intensive care unit, OR: odds ratio, SAPS II: Simplified Acute Physiology Score II.

### Predictive factors of intubation in patients treated with HFNC

In the 202 patients treated with HFNC, median age was 63 (53.2–69) and median Charlson comorbidity index was 4 (3–6) (Table 3). Underlying malignancies were acute leukemias (32.7%), lymphomas (17.3%), chronic leukemias (4.9%) and solid tumors (33.7%). Cancer was newly diagnosed in 40.6%, refractory in 43.6% and in remission in 16.8%. Allo-HSCT patients represented 17.3% of patients. Patients were neutropenic in 23.9% at ICU admission. Median SOFA score and SAPS II at ICU admission were 5 (2–7) and 42 (34–49) respectively. At HFNC initiation, median SOFA score was 5 (2–7). The number of involved pulmonary quadrants on chest X-ray was 2 (1–4). Median oxygen saturation was 94% (92–97). Fi02 was 60% (50–80) and median oxygen flow 40 L/min (40–50). Fifteen minutes after HFNC initiation, respiratory rate and Sp02 were 21 (16–29) and 96% (94–98) respectively. Median duration of HFNC since ICU admission was 0 day (0–1).

**Table 3:** Characteristics of patients treated with HFNC: Comparison of HFNC success with HFNC failure requiring intubation.

	All patients (n = 202)	No intubation (n = 104)	Intubation requirement (n = 98)	*sHR [95% CI]*	*p*
Female gender	123 (60.9%)	60 (57.7%)	63 (64.3%)	1.22 [0.81–1.84]	0.35
Age (years)	63 [53.2–69]	63 [53–69]	62.5 [54–68.7]	1 [0.9–1.02]	0.97
Charlson comorbidity index	4 [3–6]	4.5 [3–7]	4 [3–6]	0.94 [0.86–1.03]	0.19
BMI	23.9 [21.3–26.6]	24.2 [21.2–28.2]	23.6 [21.4–25.8]	0.98 [0.94–1.02]	0.31
Main underlying malignancy					
Others malignancies	23 (11.4%)	10 (9.6%)	13 (13.3%)	1 (reference)	0.367
Acute leukemias	66 (32.7%)	33 (31.7%)	33 (33.7%)	0.91 [0.48–1.73]	
Lymphomas	35 (17.3%)	14 (13.5%)	21 (21.4%)	1.14 [0.57–2.28]	
CLL/CML	10 (4.9%)	5 (4.8%)	5 (5.1%)	0.91 [0.32–2.55]	
Solid tumors	68 (33.7%)	42 (40.4%)	26 (26.5%)	0.65 [0.33–1.26]	
Allo-HSCT	35 (17.3%)	12 (11.5%)	23 (23.5%)	1.51 [0.95–2.41]	0.09
Disease status					
Newly diagnosed	82 (40.6%)	43 (41.3%)	39 (39.8%)	1 (reference)	0.72
Progression/Relapse	86 (42.6%)	42 (40.4%)	44 (44.9%)	1.01 [0.66–1.56]	
Complete/partial response	34 (16.8%)	19 (18.3%)	15 (15.3%)	0.81 [0.44–1.46]	
Documented sepsis					
Bacterial	80 (39.6%)	44 (42.3%)	36 (36.7%)	0.83 [0.55–1.25]	0.37
Including MDR bacteria	25 (12.4%)	8 (7.7%)	17 (17.3%)	1.65 [0.98–2.78]	0.08
Viral	38 (18.8%)	10 (9.64%)	28 (28.6%)	2 [1.29–3.1]	**0.003**
Fungal	30 (14.9%)	10 (9.6%)	20 (20.4%)	1.66 [1.01–2.7]	**0.05**
Fever of unknown origin	32 (15.8%)	17 (16.3%)	15 (15.3%)	1.03 [0.6–1.79]	0.91
Absence of sepsis	25 (12.4%)	20 (19.2%)	5 (5.1%)	0.31 [0.12–0.75]	**0.002**
Neutropenia at ICU admission	48 (23.9%)	23 (22.1%)	25 (25.8)	1.17 [0.74–1.85]	0.5
Severity at ICU admission					
SOFA score	5 [2–7]	4 [2–6]	5 [2.2–7]	1.07 [1.01–1.14]	**0.03**
SAPS II	42 [34–49]	40 [31–47.2]	43 [37–50]	1.03 [1.01–1.04]	**<0.001**
At HFNC initiation					
SOFA score	5 [2–7]	5 [2–6]	5 [3–8]	1.1 [1.04–1.16]	**0.003**
Nb of quadrants on X-rays	2 [1–4]	2 [1–3]	3 [2–4]	1.57 [1.33–1.85]	**<0.001**
Sp02 (%)	94 [92–97]	95 [93–98]	94 [91–96]	0.94 [0.9–0.98]	**0.009**
Fi02 (%)	60 [50–80]	55 [50–70]	70 [50–100]	1.02 [1.01–1.03]	**<0.001**
Fi02 >60%	92 (45.5%)	34 (32.7%)	58 (59.2%)	2.28 [1.52–3.41]	**<0.001**
Sp02 < 95%	102 (50.5%)	50 (48.1%)	52 (53.1%)	1.19 [1.1–2.58]	0.39
Oxygen flow (L/min)	40 [40–50]	40 [30–50]	50 [40–50]	1.04 [1.01–1.06]	**0.003**
Respiratory Rate (/min)	27 [20–32]	26 [19–30]	28 [21–34]	1.02 [1–1.05]	**0.0475**
Heart rate	110 [96–123]	109 [96–121]	111 [96–124]	1.01 [1–1.02]	0.136
Time since ICU admission (days)	0 [0–1]	0 [0–1]	[0–0]	1.00 [0.89–1.12]	0.98
Respiratory SOFA: Pa02/Fi02					
>400	1 (0.5%)	0	1 (1%)	1 (reference)	0.24
301–400	21 (10.4%)	13 (12.5%)	8 (8.2%)	0.14 [0.02–1.11]	
≤300	180 (89.1%)	91 (87.5%)	89 (90.8%)	0.20 [0.03–1.46]	
After HFNC initiation					
RR after 15 minutes	21 [16–29]	20 [15–26]	25 [17–33]	1.04 [1.02–1.06]	**<0.001**
Sp02 after 15 minutes	96 [94–98]	97 [95–98]	96 [93–98]	0.97 [0.95–0.99]	**0.03**
Sp02<95% with FiO2 = 100% after 15 minutes	80 (39.6%)	34 (32.7%)	46 (46.9%)	1.68 [1.1–2.58]	**0.02**

AlloHSCT: allogenic hematopoietic stem cell transplantation, BMI: body mass index, CLL: chronic lymphocytic leukemia, CML: chronic myeloid leukemia, Fi02: fraction of inspired oxygen, HFNC: high flow nasal cannula, ICU: intensive care unit, MDR: multi-drug resistant, RR: respiratory rate, SAPS II: simplified acute physiology score II, SOFA: sequential organ failure assessment, Sp02: oxygen saturation, sHR: subdistribution Hazard Ratio

Multivariate analysis identified 5 predictive factors of intubation. The absence of sepsis (sHR = 0.32, 95% CI = 0.12–0.74, p = 0.0087) was a protective factor regarding the intubation risk. Sp02<95% 15 minutes after HFNC initiation (sHR = 2.05, 95% CI = 1.32–3.18, p = 0.0014), the number of pulmonary quadrants involved on chest X-ray (sHR = 1.73, 95% CI = 1.46–2.06, p<0.001), Fi02>60% at HFNC initiation (sHR = 3.12, 95% CI = 2.06–4.74, p<0.001) and SAPS II at ICU admission (sHR = 1.03, 95% CI = 1.02–1.05, p<0.01) were associated with a higher risk of HFNC failure and subsequent intubation (Table 4).

**Table 4:** Multivariate analysis of prognostic factors associated with intubation.

	sHR	95% CI	p
Number of pulmonary quadrants on chest X-ray	1.73	[1.46–2.06]	**<0.001**
FiO2 >60% at HFNC initiation	3.12	[2.06–4.74]	**<0.001**
SAPS II at ICU admission	1.03	[1.02–1.05]	**<0.01**
Sp02 < 95% with FiO2 = 100% 15 minutes after HFNC initiation	2.05	[1.32–3.18]	**0.0014**
Absence of sepsis	0.32	[0.12–0.74]	**0.0087**

Allo-HSCT: allogenic hematopoietic stem cell transplantation, CI: confidence interval, HFNC: high flow nasal cannula, sHR: subdistribution Hazard ratio, SAPS II: simplified acute physiology score II.

## Discussion

Our study identified HFNC duration before intubation, disease status and severity of illness as prognostic factors of hospital mortality in ICU cancer patients with ARF requiring intubation. Risk factors of intubation were severity illness at ICU admission, FiO2 at HFNC initiation, SpO2 after HFNC initiation and sepsis.

HFNC offers interesting physiological benefits [8, 24], improves oxygenation [25], generates low-level positive airway pressure [26], reduces respiration rate [27], attenuates inspiratory resistance and supplies a constant FiO2 [28]. Moreover, it is well tolerated [29, 30] and does not increase risks of pneumonia or barotraumas [31]. The impact of HFNC duration on outcome has been poorly assessed. In a general population study, HFNC was safe and well tolerated for long periods [32]. Another retrospective study in unselected patients provided conflicting results, showing that extended use of HFNC before intubation might be harmful [33]. It was conducted on 175 patients unsuccessfully treated with HFNC. Early-intubated (<48 hours) patients had better ICU survival than late-intubated (>48 hours) patients (39.2% vs 66.7%, p = 0.001), extubation success, ventilator weaning and ventilator-free days. Recently, Dumas et al showed in a large cohort of 7736 immunocompromised patients who were intubated that time between ICU admission and intubation is a strong predictor of mortality, suggesting a detrimental effect of late initial oxygenation failure [34]. Although several studies have assessed NIV failure in immunocompromised patients [2, 17], our study is, to the best of our knowledge, the first one exploring HFNC failure in this population. Our findings revealed that early MV, before day 2, may be associated with a better outcome [35, 36]. There is a need to develop specific early warning scores to predict HFNC failure, in order to identify high-risk patients who would benefit from early intubation. Priority should move from avoiding intubation to avoiding delayed intubation. Severity of presentation, assessed by SAPS II and the number of pulmonary quadrants involved on chest X-ray, were associated with increased hospital mortality, in line with previous publications [1, 37].

To avoid late intubation, identifying prognostic factors associated with HFNC failure is important. SAPSII and severity of pulmonary extension were predictive factors of intubation. The number of involved pulmonary fields was previously identified as an early predictor of the severity of acute respiratory distress syndrome (ARDS) in hematology patients [23, 38]. Fi02 at HFNC initiation was another significant factor. Similarly, the degree of hypoxemia (PaO2/FiO2 after 1 hour of NIV) was associated with NIV failure [10, 18, 39]. Predictors of NIV failure have been summarized in an easy to use mnemonic “HACOR” score (Heart rate, Acidosis, Consciousness, Oxygenation, Respiratory rate) to identify patients at high risk for NIV failure [40]. Similarly, further studies are needed to confirm the determinants of HFNC failure.

Due to its ease of application, non-evidence-based use of HFNC has spread to non-ICU wards [41]. To consider safely HFNC outside the ICU, the identification of patients at low risk of intubation is essential. Exploratory studies are needed in this context.

So far, strategies to improve survival in hypoxemic patients with ARF relied on different oxygenation options [3, 7]. More than ventilation strategy, the stronger prognostic features were mechanical ventilation and absence of ARF etiology [2]. This is why we focused in patients requiring intubation in our study, exploring the impact of HFNC in this population. Future researches should focus on optimal timing of ICU admission, personalizing an appropriate oxygenation strategy according to situations [42] and selecting the most relevant diagnostic strategy [2, 43]. Collaboration between onco-hematologists and intensivists is crucial [6]. ARF with undetermined etiology impacts outcome, as previously described [3]. A timely and accurate diagnostic strategy that takes into account characteristics of the underlying malignancy, immunosuppression, respiratory symptoms and radiologic pattern [2, 3, 44, 45]. In addition, preventing ICU acquired events from both MV and underlying impairment of immunological functions will also be challenging [1, 4, 46].

We acknowledge some limitations in our study. First, its retrospective nature is intrinsically susceptible to have selection bias. However all biological and medical settings were prospectively collected. Secondly, this is a monocentric study in a highly specialized cancer center. Generalization of our results may therefore be analyzed with caution. Third, we measured respiratory distress in our study and not dyspnea. Recent data from the GRRROH showed that dyspnea was frequent and intense in patients receiving NIV for ARF and was associated with a higher risk of NIV failure and poorer outcome [47]. Another study showed that the magnitude of inspiratory effort relief as assessed by esophageal pressure variation within the first 2 hours of NIV was an early and accurate predictor of NIV outcome at 24 hours [48]. Dyspnea would be of great interest to assess in future studies. Lastly, our study was unfortunately not designed to explore ROX index, the ratio of oxygen saturation as measured by pulse oximetry/Fi02 to respiratory rate, recently reported in both immunocompetent and immunocompromised patients. Recent data showed that in patients with pneumonia with ARF treated with HFNC, ROX index could help to identify patients with low and high risk for intubation in a 2-year multicenter prospective observational cohort study [49]. Lemiale et al. recently reported the performance of the ROX index to predict intubation in immunocompromised patients receiving HFNC for ARF. A ROX index greater than 4.88 appeared to have a poor ability to predict intubation, although it remained highly associated with the risk of intubation and may be useful to stratify such risk in future studies [50].

## Conclusions

Duration of HFNC may be predictive of mortality in ARF cancer patients requiring intubation after unsuccessful HFNC. There is a need to develop specific early warning scores to predict HFNC failure in order to identify high-risk patients who might benefit from early intubation. Whatever the technique of oxygenation used, day-to-day decisions must strive to avoid delayed intubation and identify ARF etiology.
